# Supplementary material for: Chimeric reference panels for genomic imputation
Source: Genetics. 2025 Oct 1;232(1):iyaf212. doi: 10.1093/genetics/iyaf212 (PMC12774824; doi:10.1093/genetics/iyaf212)
Supplement: iyaf212_Supplementary_Data [file iyaf212_supplementary_data.pdf]

Supporting information

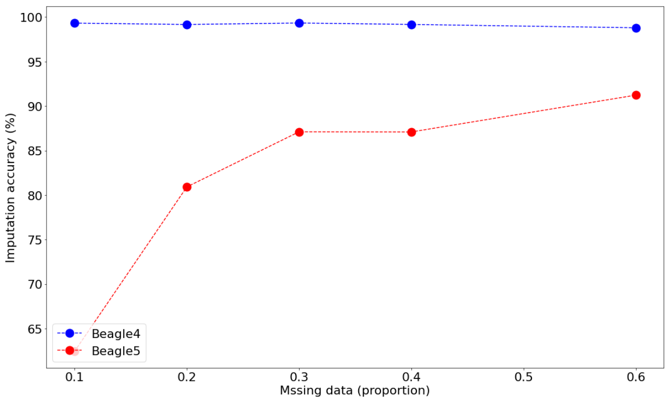

Supplementary Figure 1 Significant performance difference in the imputation of small external reference panel size (50 individuals) by Beagle4 and Beagle5.

Comparative analysis of Beagle versions and small sample size considerations

In our methodological evaluation, we observed a significant performance difference between Beagle4 and the more recent Beagle5 when applied to small reference panels (Supplementary Figure 1). This phenomenon warranted deeper investigation, as it directly impacts implementation decisions for non-model organism research where reference sample sizes are inherently constrained.

Through correspondence with the principal developer of Beagle5, we received confirmation that the algorithm's statistical framework was optimized specifically for large-scale human genomic studies with substantial reference panels. The Beagle5 algorithm implements sophisticated haplotype clustering algorithms that demonstrate substantially improved computational efficiency and imputation accuracy when supplied with reference panels comprising hundreds to thousands of individuals. However, these same algorithmic optimizations create performance instability when the reference panel falls below certain statistical thresholds.

We determined that Beagle4 (v 4.1) maintains robust performance even with modest reference panel sizes (25-50 individuals), making it appropriate for integration with Retriever's chimeric panel. This finding has significant implications for research communities working with non-model organisms, as it suggests that algorithm selection should be guided by dataset characteristics rather than simply defaulting to the most recent software iteration. Ongoing technical discussions with the Beagle development team aim to characterize the precise statistical thresholds and parameterization adjustments that might enable Beagle5 to function effectively with smaller reference panels. Future releases of Retriever will incorporate these methodological refinements as they become available, potentially extending the computational advantages of newer imputation algorithms to non-model organism research.

Algorithm details

Retriever constructs the chimeric reference panel by iteratively scanning the genomic positions with a sliding window. For each window, the algorithm extracts genotype data, removes individuals (columns) with missing genotypes (coded as -3), a negative number was chosen to represent missing genotypes to prevent confusion with present genotype calls, and then checks whether the number of remaining (complete) individuals meets the user-defined requirement. If the count is insufficient, the window is reduced until the required number is achieved or the minimum window size is reached; if even the smallest window does not yield enough complete data, an error is raised. Otherwise, a random subset of individuals from the complete data is selected to form a reference "bucket". These buckets, extracted from non-overlapping windows, are then concatenated chronologically to assemble the final chimeric reference panel.

The following pseudocode outlines the method:

Algorithm 1 Chimeric Reference Panel Construction

```
procedure ExtractReference(Genotype matrix (G), genomic positions (P), required individuals (k), window size (W))
    i ← 0
    Initialize reference genotype matrix R ← {} and position list R_P ← {}
    while i < |P| do
        Identify index a such that P[a] is the smallest position with P[a] ≥ P[i] + W;
        if no such a exists, set a ← |P| then
            Extract block B ← G[i : a + 1, :] corresponding to positions P[i] to P[a]
            Remove columns in B where missing data (−3) occurs, yielding B′
            if number of columns in B′ < k then
                Reduce a ← a − 1 and update B′
                if i = a or B′ still has less than k columns then
                    Raise error: "Insufficient complete genotypes for window at position P[i]"
            else
                Randomly sample k columns from B′ to form the reference bucket B_R
                Append B_R to R and record positions P[i : a + 1] in R_P
                Set i ← a + 1                ▷ Advance to next non-overlapping window
    return R, R_P
```

The final chimeric reference panel is obtained by concatenating all buckets B_R extracted across the genome:

$$R \leftarrow \bigcup_{\text{window}} B_R.$$

The performance of this approach depends critically on:

- The initial window size W (defaulting to 1 kb, or a user-defined value), which determines the genomic span considered in each iteration.
- The minimum window size (e.g., two bp), ensuring that data is extracted even in regions with high missingness.
- The required number k of individuals per window, which balances the need for sufficient diversity with the constraint of available complete data.

By using this strategy, Retriever leverages the complementary distribution of missing data across samples while preserving the local haplotype structure necessary for effective downstream genotype imputation.
